# Cardio-oncology-relevant programmes in the cervical cancer tumour microenvironment: an exploratory multi-omics and endothelial-cell study

**DOI:** 10.3389/fcvm.2026.1917892

**Published:** 2026-09-23

**Authors:** Rongrong Cai, Suqin Shen, Chunyan Gu

**Affiliations:** Department of Gynecology, Nantong Haimen People’s Hospital, Nantong, China

**Keywords:** anti-angiogenic therapy, bevacizumab, cardio-oncology, cardiotoxicity risk prediction, cardiovascular toxicity, cervical cancer, immune checkpoint inhibitor, multi-omics

## Abstract

**Background:**

Bevacizumab and immune-checkpoint inhibitors (ICIs) improve outcomes in advanced cervical cancer but may cause cardiovascular toxicity (CVT). Public cervical-cancer transcriptomic datasets do not contain cardiac tissue or adjudicated CVT outcomes; consequently, tumour-microenvironment (TME) expression cannot by itself establish a cardiac susceptibility mechanism. We reanalysed the available data to ask the narrower question of whether cervical tissues contain angiogenic, endothelial, inflammatory, T-cell-exhaustion, and TGF-*β*/fibrotic programmes that are biologically relevant to cardio-oncology.

**Methods:**

All nine single-cell RNA-sequencing specimens in GSE208653 were analysed: two HPV-negative healthy cervix, two HPV-positive histologically normal cervix, two high-grade squamous intraepithelial lesions, two squamous-cell carcinomas, and one adenocarcinoma. After prespecified quality control, 74,457 cells were integrated with Harmony and annotated into broad lineages. Prespecified module scores and four ligand–receptor expression-product scores were aggregated by biological specimen; malignant versus non-malignant differences were evaluated with exact specimen-label permutation tests and Benjamini–Hochberg correction. Four GSE208654 Visium count matrices were analysed descriptively at spot level. Coexpression among the five programmes was assessed in 304 TCGA-CESC primary tumours. qRT-PCR summary data from HUVEC (ATCC PCS-100-010) and primary human aortic endothelial cells (HAEC; ATCC PCS-100-011) were reinterpreted as endothelial pharmacology, not cardiac validation.

**Results:**

The scRNA-seq atlas contained 32 clusters spanning epithelial, immune, endothelial, and stromal lineages. None of the five specimen-level programmes differed significantly between malignant and non-malignant specimens after multiple-testing correction (all q ≥ 0.119). Likewise, none of the VEGFA–KDR, IL1B–IL1R1, TGFB1–TGFBR2, or LGALS9–HAVCR2 expression-product scores was significant (all q ≥ 0.143). The four spatial specimens showed heterogeneous spot-level expression but, with one specimen per histology and no slide coordinates in the supplied matrices, did not support group-level spatial inference. TCGA tumours showed coordinated intratumoural expression, strongest between angiogenesis and TGF-*β*/fibrosis (Spearman *ρ*=0.707; q = 2.24 × 10⁻46), but TCGA-CESC contained no CVT endpoint. VEGFA increased KDR, ICAM1, VCAM1, and EDN1 mRNA in HUVEC, and bevacizumab attenuated these changes; IL-1*β* induced inflammatory transcripts in HAEC, and anakinra attenuated IL6, TNF, and CXCL8.

**Conclusions:**

Cervical tissues contain cardio-oncology-relevant transcriptional programmes, but the available data neither demonstrate cardiac expression nor identify bevacizumab- or ICI-induced CVT susceptibility. The endothelial experiments show expected pathway responsiveness and pharmacological blockade, not treatment-induced cardiotoxicity. Prospective analyses must compare CVT-positive and CVT-negative treated patients using matched clinical outcomes and, ideally, blood or cardiac-relevant biospecimens.

## 1 Introduction

Cervical cancer remains a major cause of cancer morbidity and mortality in women worldwide, with the greatest burden occurring in regions where screening, human-papillomavirus (HPV) vaccination, and access to multimodality treatment remain uneven (1). Persistent infection with oncogenic HPV initiates a prolonged sequence from infected but histologically normal epithelium through high-grade squamous intraepithelial lesions (HSIL) to invasive carcinoma. Although many patients with early disease can be cured by surgery or chemoradiotherapy, recurrent, persistent, or metastatic cervical cancer has historically carried a poor prognosis. Improving systemic therapy has therefore become a central clinical priority.

The treatment landscape has changed substantially during the past decade. GOG 240 established that adding bevacizumab to platinum-based chemotherapy improves overall survival in advanced cervical cancer, with the final analysis confirming sustained benefit while also documenting treatment-related vascular adverse events (2, 3). Immune-checkpoint inhibitors (ICIs) subsequently broadened systemic options: pembrolizumab improved outcomes when added to first-line chemotherapy with or without bevacizumab, and cemiplimab improved survival after progression on platinum-containing therapy (4, 5). More recently, the BEATcc trial showed that adding atezolizumab to bevacizumab plus chemotherapy further improved progression-free and overall survival (6). As effective regimens are administered for longer and combine agents with different toxicity profiles, preservation of cardiovascular health has become increasingly relevant to treatment continuity and long-term outcome (7).

Bevacizumab neutralises vascular endothelial growth factor A (VEGFA), a central regulator of angiogenesis and endothelial survival. Its established adverse-event spectrum includes new or worsened hypertension, proteinuria, arterial thromboembolic events, haemorrhage, and, less frequently, heart failure or myocardial ischaemia (2, 3, 7). VEGF signalling through VEGFR2/KDR maintains endothelial nitric-oxide production, vascular tone, microvascular integrity, and renal glomerular homeostasis (8–11). Systemic VEGF inhibition can therefore increase peripheral resistance and alter renal sodium handling and microvascular function. Importantly, this pharmacological mechanism concerns the consequences of inhibiting physiological VEGF signalling throughout the circulation. High VEGFA expression inside a tumour is not equivalent to increased VEGF signalling in cardiac tissue and does not, without outcome-linked evidence, identify an individual patient who will develop bevacizumab-associated cardiovascular toxicity (CVT).

ICI-related cardiovascular adverse events are biologically and clinically distinct. They include myocarditis, pericardial disease, arrhythmias, conduction disturbance, heart failure, vasculitis, and potentially accelerated atherosclerotic events (7, 12–17). ICI myocarditis is uncommon but can be fulminant, particularly when recognised late or accompanied by myositis, myasthenic features, ventricular arrhythmia, or high-grade conduction block (12–14). Direct studies in affected patients and experimental models support a T-cell-dominant process, including expansion of activated or clonally related cytotoxic CD8 + populations (16). Nevertheless, the inciting antigens, determinants of organ tropism, and reasons only a minority of exposed patients develop myocarditis remain incompletely defined (17). These uncertainties make comparisons between treated CVT-positive and treated CVT-negative patients essential for susceptibility research.

The cervical tumour microenvironment (TME) is heterogeneous and changes during HPV-associated malignant progression. Epithelial cells coexist with T and NK cells, myeloid populations, endothelial cells, pericytes, fibroblasts, and cancer-associated fibroblasts (CAFs), producing angiogenic, inflammatory, immune-suppressive, and extracellular-matrix programmes (18–20). Single-cell RNA sequencing and spatial transcriptomics can resolve which cervical-tissue compartments express ligands, receptors, and pathway genes that are obscured in bulk averages. These approaches are valuable for describing tumour biology and for generating treatment-relevant hypotheses. They do not, however, establish protein secretion, physical cell contact, systemic exposure, signalling in distant organs, or clinical toxicity.

That evidentiary boundary is particularly important for axes such as VEGFA–KDR, IL1B–IL1R1, TGFB1–TGFBR, and LGALS9–HAVCR2. Detecting both components in cervical tissue shows that the corresponding transcripts are present in that tissue. It does not show that the same axis is increased in the myocardium, nor that it separates patients who will and will not develop CVT. CAFs are tumour-stromal cells and cannot be treated as cardiac resident cells. Similarly, a cervical exhausted-T-cell programme cannot be assumed to represent the clonally expanded circulating or myocardial T-cell populations observed in ICI myocarditis. Tumour transcriptomics may ultimately contribute candidate predictors, but such predictors must be tested against prospectively ascertained outcomes.

The source analysis selected one specimen per disease category, pooled cells across specimens, labelled simple ligand–receptor expression products as communication probabilities, and used tumour-only TCGA data to construct a CVT-risk framework. We therefore performed a complete reanalysis using every GSE208653 specimen and the biological specimen as the inferential unit. We prespecified five broad programmes and four transparent ligand–receptor expression-product summaries, treated GSE208654 as descriptive spot-level data, and limited TCGA-CESC to intratumoural coexpression. The objective was not to claim a cardiac mechanism or discover a clinical CVT biomarker, but to determine which cardio-oncology-relevant programmes are detectable in cervical tissue, quantify their specimen-level uncertainty, and define the additional evidence required for causal or predictive inference.

## 2 Methods

### 2.1 Study design and evidentiary scope

This was a secondary analysis of public cervical-tissue transcriptomes plus a reinterpretation of previously generated endothelial-cell qRT-PCR summary data. No analysed public cohort contained anticancer-treatment exposure, cardiac biospecimens, adjudicated CVT, or a CVT-positive/CVT-negative grouping. The primary inferential comparison was therefore malignant cervical specimens (two SCC and one ADC) versus the six non-malignant cervical specimens (healthy, HPV-positive normal, and HSIL); this comparison concerns cervical-tissue programmes only.

Cells or spatial spots were used to estimate specimen-level summaries but were not treated as independent patients. All pathway names were neutral biological labels rather than ‘CVT modules’ or ‘risk scores’. No ROC curve, clinical predictor model, or CVT-risk classification was constructed.

### 2.2 Data sources and specimen inclusion

We used every count matrix available in GEO series GSE208653 (18). No specimen was selected for representativeness. The exact accessions, source labels, and retained cell counts are shown in Table 1.

**Table 1:** All GSE208653 specimens included in the revised single-cell analysis. HSIL, high-grade squamous intraepithelial lesion; QC, quality control.

GSE208653 condition	GEO accessions	Specimens	Cells after QC
HPV-negative healthy cervix	GSM6360680; GSM6360681	2	18,424
HPV-positive normal histology	GSM6360682; GSM6360683	2	21,560
HSIL	GSM6360684; GSM6360685	2	12,333
Squamous-cell carcinoma	GSM6360686; GSM6360687	2	13,958
Adenocarcinoma	GSM6360688	1	8,182

GSE208654 provided four 10x Visium gene-count matrices: normal cervix (GSM6360689), HSIL (GSM6360690), SCC (GSM6360691), and ADC (GSM6360692), one specimen per condition (18). TCGA-CESC primary-tumour STAR-TPM profiles were accessed from the UCSC Xena GDC hub; 304 primary tumours with the selected genes were retained.

### 2.3 Single-cell quality control, integration, and annotation

Matrices were imported with Scanpy 1.12.3 following standard single-cell quality-control principles (21, 22). Cells were retained if they expressed 200–5,999 genes and had <20% mitochondrial reads; genes detected in fewer than three cells were removed. Counts were normalised to 10,000 per cell and log1p transformed. We selected 3,000 highly variable genes with specimen as the batch key, scaled the data, calculated 40 principal components, and integrated specimens with Harmony 2.0.0 (23). A 15-nearest-neighbour graph used the first 30 Harmony components. UMAP was used for visualisation and Leiden clustering used resolution 0.7 (24). The fixed random seed was 20260731.

Clusters were assigned to 11 broad lineages by the highest average expression of prespecified canonical marker panels: epithelial, T cell, NK cell, B cell, plasma cell, myeloid, endothelial, fibroblast/CAF, pericyte/smooth-muscle cell, mast cell, and proliferating. Broad annotation was chosen because the small number of specimens does not support reliable claims about fine subtypes.

### 2.4 Prespecified programme scoring and specimen-level inference

Five compact gene panels were defined before group testing: angiogenesis, endothelial activation, inflammation, T-cell exhaustion, and TGF-*β*/fibrosis. Genes were selected from relevant MSigDB Hallmark concepts, gene-set methodology, and established T-cell-exhaustion literature (25–27). These panels were used as descriptive summaries, not validated clinical biomarkers.

For each cell, a programme score was the mean log-normalised expression of available member genes. Scores were then averaged within each biological specimen. To improve biological specificity, endothelial activation was summarised within endothelial cells, inflammation within myeloid cells, T-cell exhaustion within T cells, and TGF-*β*/fibrosis within fibroblast/CAF cells; angiogenesis was summarised across all cells. Malignant-versus-non-malignant differences were tested with an exact two-sided permutation test over all assignments of three of nine specimens to the malignant group. Benjamini–Hochberg correction was applied across the five programmes. Effect sizes are malignant minus non-malignant differences on the log-normalised score scale.

### 2.5 Ligand–receptor expression-product summaries

Four biologically relevant ligand–receptor pairs were evaluated in prespecified cervical-TME sender/receiver contexts: fibroblast/CAF VEGFA to endothelial KDR; myeloid IL1B to endothelial IL1R1; myeloid TGFB1 to fibroblast/CAF TGFBR2; and myeloid LGALS9 to T-cell HAVCR2. Within each specimen, the score was the mean ligand expression in the sender lineage multiplied by mean receptor expression in the receiver lineage, provided both lineages contained at least 20 cells.

These scores are simple expression products. They do not model protein abundance, secretion, physical proximity, receptor occupancy, directionality, or signalling probability and are not presented as CellChat or CellPhoneDB results. The same exact permutation framework compared malignant and non-malignant specimens, with Benjamini–Hochberg correction across four axes. CAF-related results were explicitly restricted to the cervical TME and were not extrapolated to myocardium.

### 2.6 Spot-level analysis of Gse208654

Visium count matrices were filtered to retain spots with at least 100 detected genes and <25% mitochondrial reads, then normalised and log1p transformed. After filtering genes detected in fewer than three spots, 2,000 batch-aware highly variable genes were used for PCA, neighbours, UMAP, and Leiden clustering. Programme scores were calculated per spot. Spatial transcriptomic methods can relate gene expression to tissue position when coordinates and histology are available (28); however, the supplied matrices did not include those components. UMAP therefore represents transcriptomic similarity among spots rather than physical tissue location. With one specimen per histology, no inferential comparison among disease groups was performed.

### 2.7 TCGA-CESC tumour coexpression

For 304 TCGA-CESC primary tumours (29, 30), selected-gene expression was analysed as log2(TPM+1). Each programme score was the within-tumour mean of its available genes. Pairwise Spearman correlations among the five programmes were calculated and corrected across ten pairs by Benjamini–Hochberg. Because TCGA-CESC lacks adjudicated CVT, these analyses estimate coexpression among tumour programmes only. Tumours were not labelled as high or low CVT risk.

### 2.8 Endothelial-cell experiments and qRT-Pcr

The catalogue identities were checked against ATCC. PCS-100-010 is primary human umbilical-vein endothelial cells (HUVEC), and PCS-100-011 is primary human aortic endothelial cells (HAEC), not AC16 cardiomyocytes. Both are endothelial models. Cells were maintained in Vascular Cell Basal Medium (ATCC PCS-100-030) supplemented with Endothelial Cell Growth Kit-VEGF (ATCC PCS-100-041) according to the supplier's instructions. No cardiomyocyte or cardiac-injury validation was inferred, and authentication or mycoplasma status was not independently evaluated in this reanalysis.

For the VEGF experiment, HUVEC were treated for 24 h with vehicle, recombinant human VEGFA (50 ng/mL), or VEGFA plus bevacizumab (2.5 μg/mL); KDR, ICAM1, VCAM1, and EDN1 mRNA were measured. For the inflammatory experiment, HAEC were treated for 24 h with vehicle, recombinant human IL-1*β* (10 ng/mL), or IL-1*β* plus anakinra (100 ng/mL); IL1R1, NFKB1, IL6, TNF, and CXCL8 mRNA were measured.

As reported in the source methods, total RNA was isolated with TRIzol, reverse-transcribed with PrimeScript RT Reagent Kit with gDNA Eraser, and quantified using TB Green Premix Ex Taq II on a QuantStudio 7 Flex. Relative expression was normalised to GAPDH by the 2−*ΔΔ*Ct method (31). The source manuscript reported three biological replicates with technical triplicates. However, only plotted or tabulated mean ± SEM values were available for independent review; replicate-level Ct data, amplification quality-control records, and primer sequences were unavailable. Accordingly, the revised results present the available summaries descriptively and do not reconstruct individual points or rederive P values.

## 3 Results

### 3.1 An all-specimen cervical single-cell atlas

All nine GSE208653 specimens were included. Of 83,959 input cell barcodes, 74,457 passed quality control; 18,494 genes remained after filtering. Retained cells per specimen ranged from 5,467 to 14,906. Harmony-integrated clustering yielded 32 clusters assigned to 11 broad lineages. The UMAP displayed epithelial, immune, endothelial, and stromal compartments, while cell-type proportions varied substantially between specimens within the same condition (Figure 1). This between-specimen variation motivated specimen-level rather than cell-level inference.

**Figure 1:**
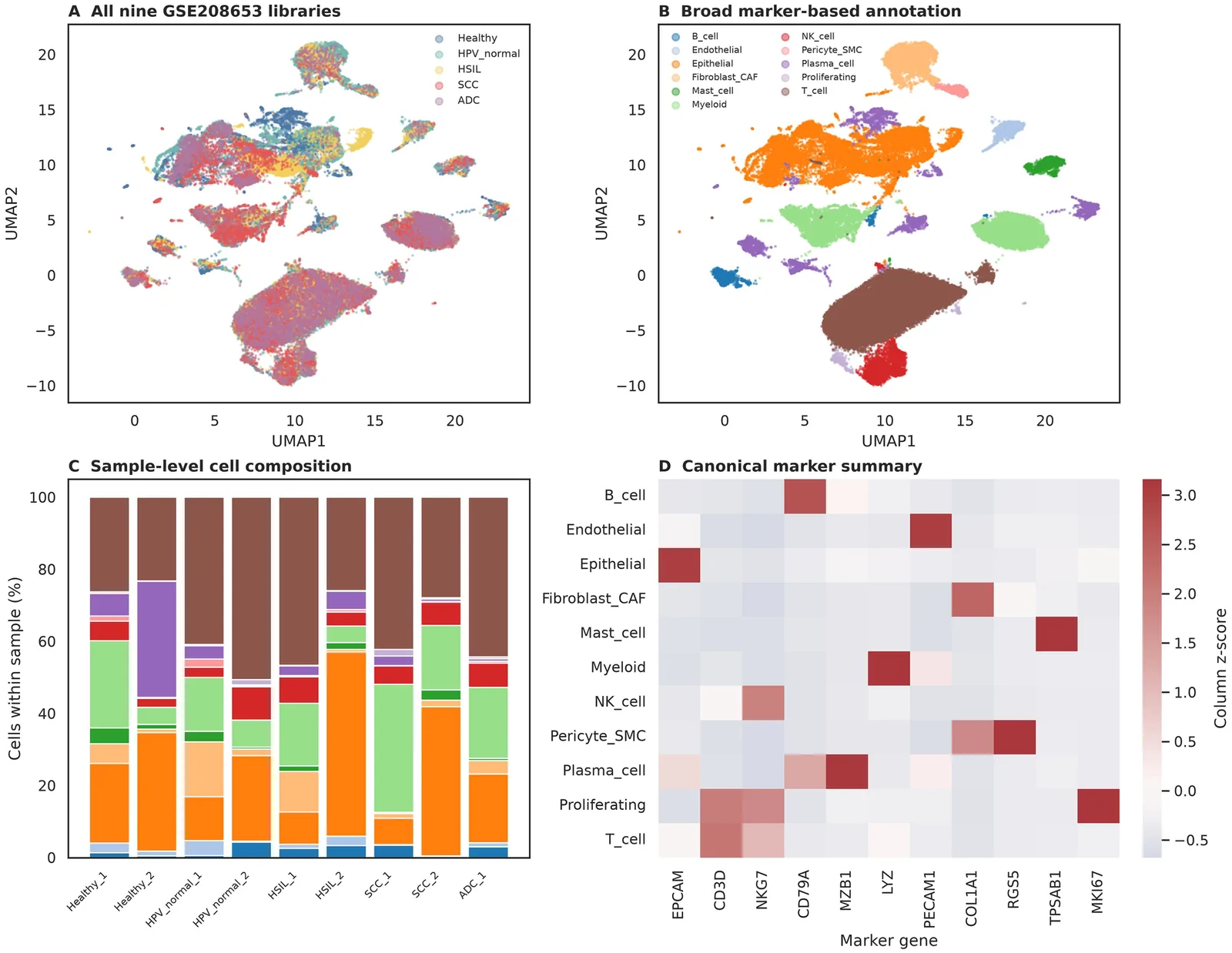
All-specimen single-cell atlas of GSE208653. **(A)** UMAP of 74,457 cells coloured by cervical-tissue condition. **(B)** UMAP coloured by broad marker-panel lineage. **(C)** Specimen-level cell-lineage composition. **(D)** Heatmap of lineage marker expression. UMAP is a visualisation and not an inferential test.

### 3.2 No prespecified programme was significant at specimen level

All five mean programme scores were numerically higher in the three malignant specimens than in the six non-malignant specimens, but none met the prespecified false-discovery threshold (Figure 2). The malignant-minus-non-malignant differences were 0.009 for angiogenesis (exact *P* = 0.583; q = 0.690), 0.162 for endothelial activation (*P* = 0.333; q = 0.556), 0.336 for inflammation (*P* = 0.190; q = 0.476), 0.082 for TGF-*β*/fibrosis (*P* = 0.690; q = 0.690), and 0.147 for T-cell exhaustion (*P* = 0.0238; q = 0.119). Thus, the smallest nominal P value did not survive correction, and the data do not support a claim that these programmes are significantly upregulated in malignant cervical tissue.

**Figure 2:**
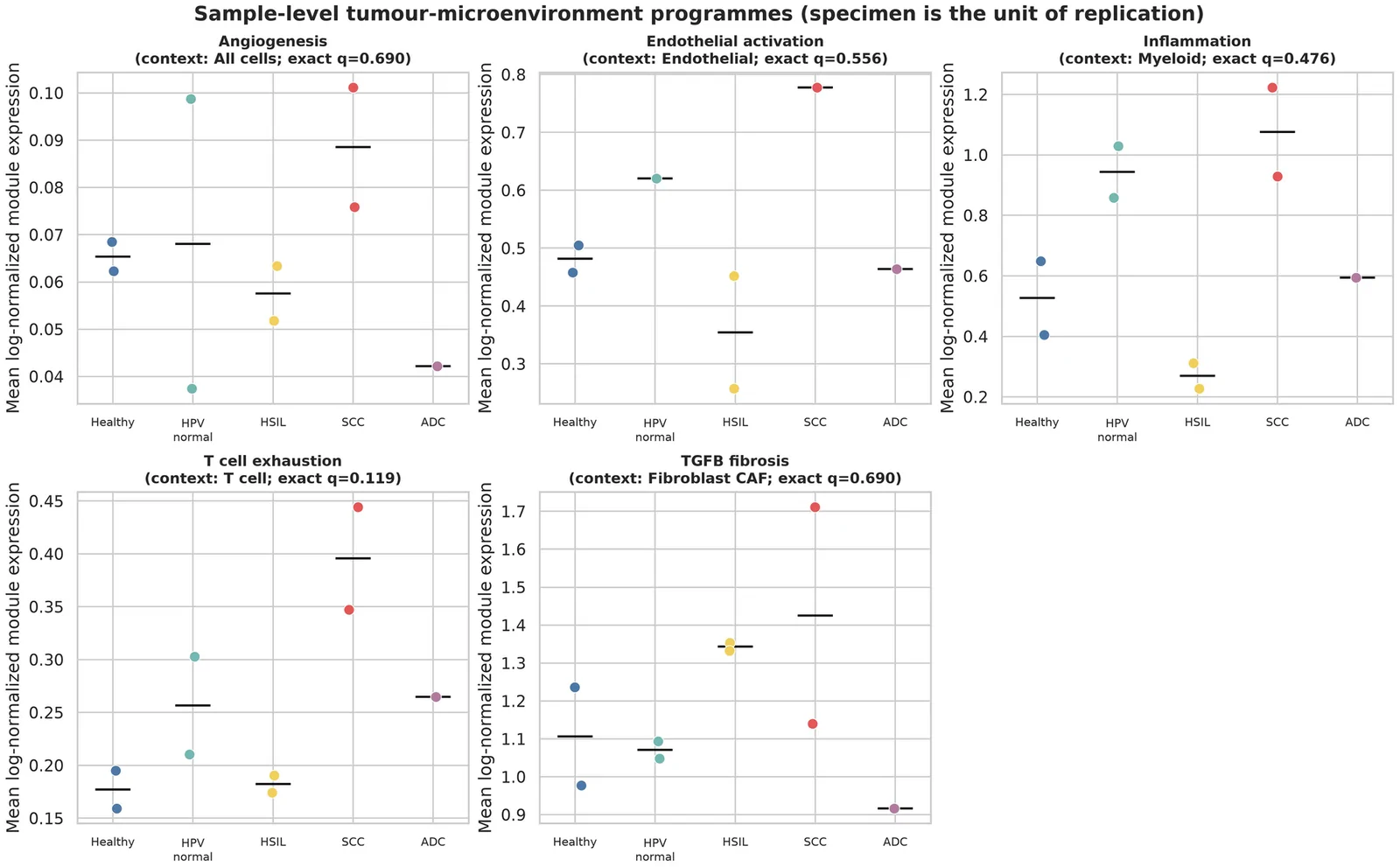
Specimen-level programme scores. Points are biological specimens; lines connect specimens only to aid Reading. Exact two-sided malignant-versus-non-malignant permutation tests were corrected across five programmes. No programme met q < 0.05.

### 3.3 Cervical-TME ligand–receptor expression products were not significant

The four expression-product summaries were detectable in subsets of specimens, confirming coexpression of the relevant ligand and receptor transcripts in the specified cervical-TME lineages (Figure 3). None differed significantly between malignant and non-malignant specimens: VEGFA–KDR effect 0.029 (*P* = 0.905; q = 0.905), IL1B–IL1R1 effect 0.129 (*P* = 0.810; q = 0.905), TGFB1–TGFBR2 effect −0.049 (*P* = 0.310; q = 0.619), and LGALS9–HAVCR2 effect 0.081 (*P* = 0.0357; q = 0.143). These results are confined to cervical tissue. In particular, fibroblast/CAF-to-endothelial VEGFA–KDR expression does not imply a CAF population or the same interaction in cardiac tissue.

**Figure 3:**
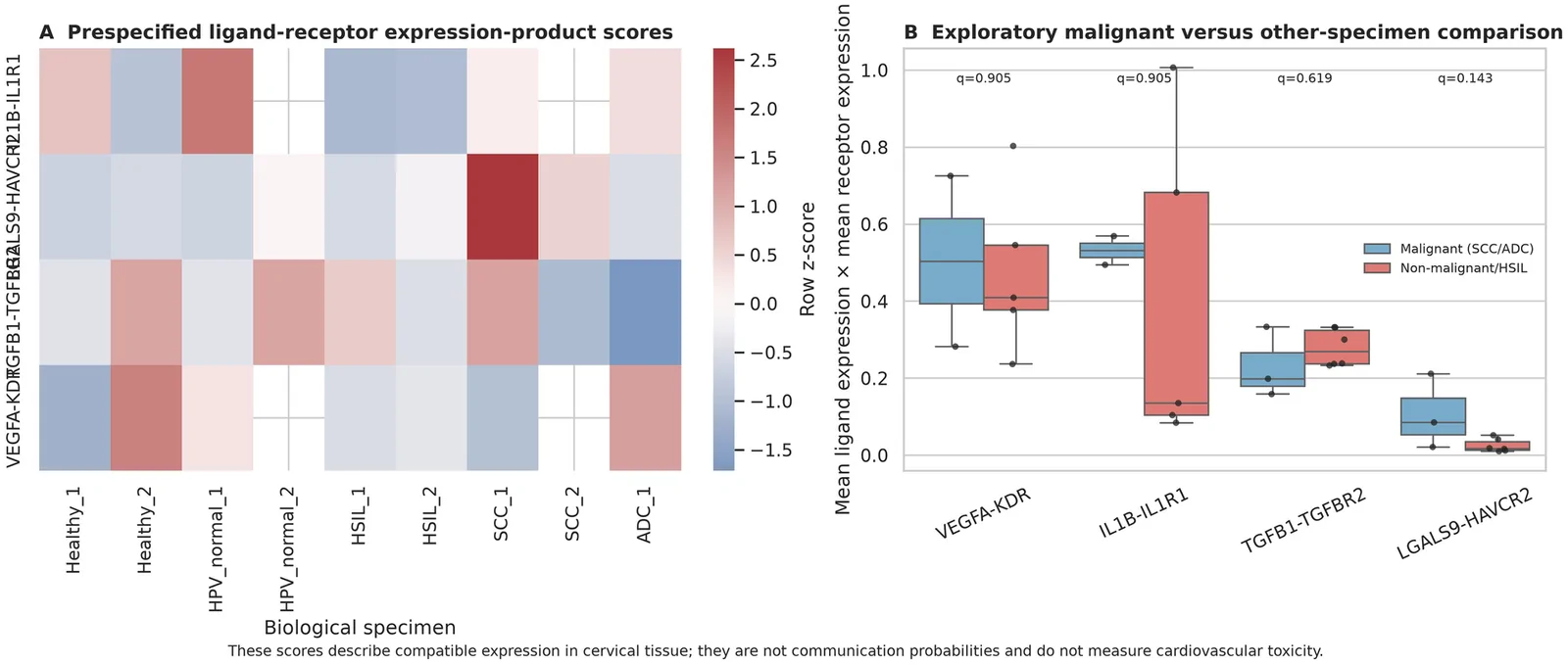
Cervical-TME ligand–receptor expression-product summaries. **(A)** Specimen-b*y*-axis heatmap. **(B)** Specimen-level scores for the four prespecified axes and exact-test q values. Scores are products of lineage-level transcript means, not communication probabilities, and do not represent cardiac tissue.

### 3.4 Spatial count matrices provide descriptive spot-level support only

After quality control, 4,852 spots remained: 938 normal, 565 HSIL, 1,757 SCC, and 1,592 ADC. Transcriptomic UMAPs showed heterogeneity across and within specimens, and VEGFA, IL1B, and TGFB1 were expressed in subsets of spots (Figure 4). Because each histology was represented by one specimen and physical slide coordinates were unavailable in the supplied matrices, these findings neither establish spatial colocalisation nor support a malignant-versus-normal statistical claim.

**Figure 4:**
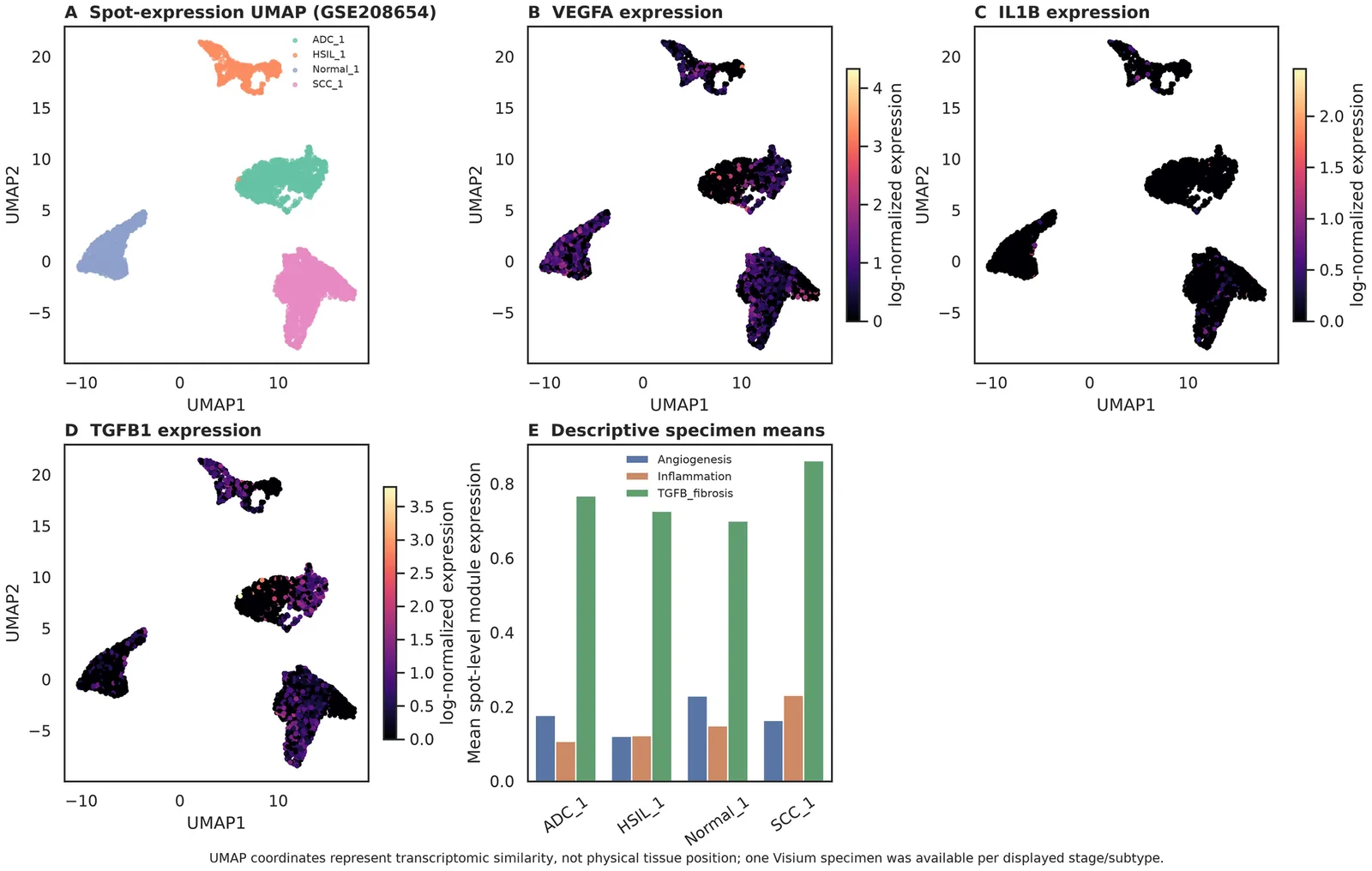
Descriptive GSE208654 spot-level analysis. **(A)** Transcriptomic UMAP of 4,852 spots by specimen. **(B–D)** VEGFA, IL1B, and TGFB1 expression on the transcriptomic UMAP. **(E)** Mean spot-level module expression by specimen. Physical slide coordinates were unavailable; one specimen per histology precludes group inference.

### 3.5 TCGA-CESC demonstrates tumour coexpression, not CVT prediction

Across 304 TCGA-CESC primary tumours, nine of ten programme pairs had positive nominal correlations and eight remained significant after correction (Figure 5). The strongest association was angiogenesis with TGF-*β*/fibrosis (Spearman *ρ*=0.707; q = 2.24 × 10⁻46), followed by endothelial activation with TGF-*β*/fibrosis (*ρ*=0.563; q = 4.19 × 10⁻26), endothelial activation with inflammation (*ρ*=0.518; q = 8.59 × 10⁻22), and endothelial activation with T-cell exhaustion (*ρ*=0.498; q = 4.33 × 10⁻20). Angiogenesis and T-cell exhaustion were not significantly correlated (*ρ*=0.082; q = 0.155), and the T-cell-exhaustion/TGF-*β* pair narrowly missed the corrected threshold (*ρ*=0.114; q = 0.052). These correlations show coordination within tumour transcriptomes only; no patient was classified by CVT risk.

**Figure 5:**
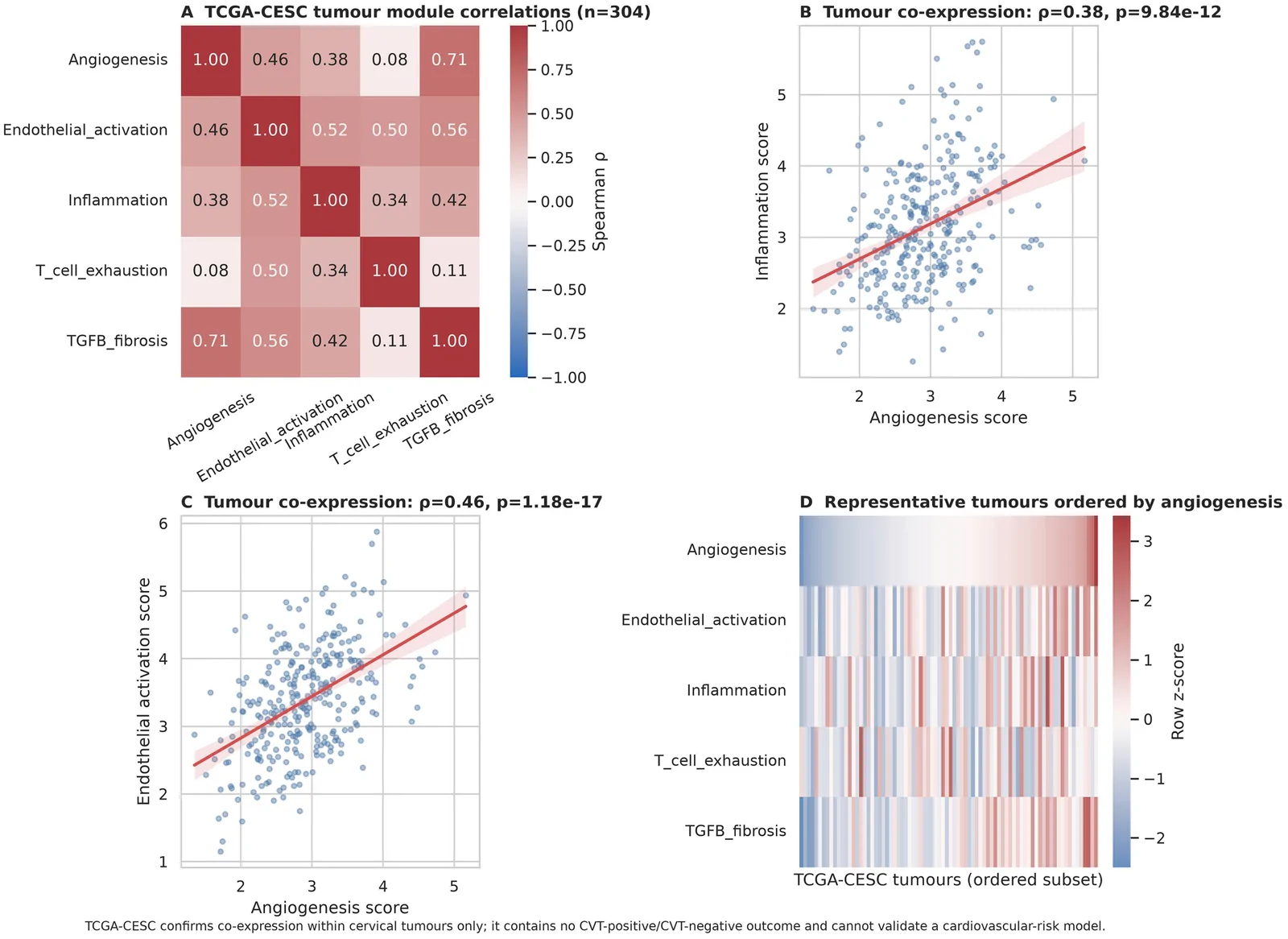
TCGA-CESC tumour coexpression. **(A)** Spearman correlation matrix for five selected-gene programme scores in 304 primary tumours. **B, C** Representative tumour-level associations. **D** Programme-score distributions across tumours ordered by angiogenesis. These are tumour coexpression patterns; TCGA-CESC has no adjudicated CVT outcome.

### 3.6 Endothelial experiments show pathway responsiveness and blockade

In HUVEC, VEGFA increased the supplied mean relative mRNA values for KDR (3.21 ± 0.38), ICAM1 (4.68 ± 0.62), VCAM1 (3.94 ± 0.51), and EDN1 (2.83 ± 0.34) compared with control (approximately 1.0). Adding bevacizumab reduced the respective summaries to 1.42 ± 0.21, 1.79 ± 0.28, 1.63 ± 0.24, and 1.31 ± 0.19 (Figure 6A). The direction of this result is consistent with on-target neutralisation of exogenous VEGFA. It does not show that VEGFA expression causes bevacizumab-associated CVT; nor does it model the loss of systemic endothelial VEGF homeostasis during chronic treatment.

**Figure 6:**
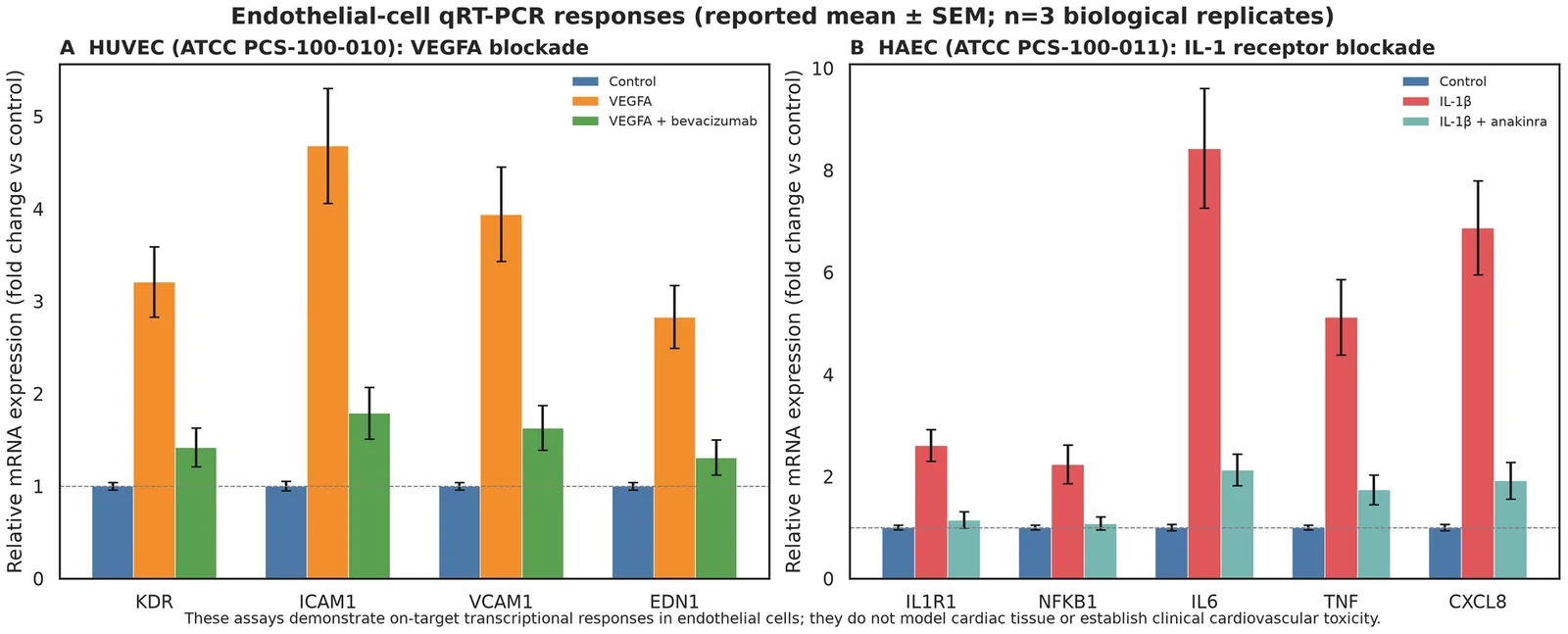
Corrected interpretation of endothelial-cell qRT-PCR summaries. **(A)** HUVEC (ATCC PCS-100-010) after vehicle, VEGFA, or VEGFA plus bevacizumab. **(B)** HAEC (ATCC PCS-100-011), not AC16 cardiomyocytes, after vehicle, IL-1β, or IL-1β plus anakinra. Bars show supplied mean ± SEM; raw Ct values were unavailable and individual points or P values were not reconstructed. The experiments show pathway responsiveness and blockade, not cardiac toxicity.

In HAEC—not AC16 cardiomyocytes—IL-1*β* increased the supplied mean values for IL1R1 (2.61 ± 0.31), NFKB1 (2.24 ± 0.38), IL6 (8.43 ± 1.17), TNF (5.12 ± 0.74), and CXCL8 (6.87 ± 0.92). With anakinra, IL6, TNF, and CXCL8 were 2.13 ± 0.31, 1.74 ± 0.29, and 1.92 ± 0.36, respectively (Figure 6B). This is compatible with IL-1-receptor-dependent inflammatory signalling in aortic endothelial cells. It is not evidence of cardiomyocyte injury or ICI myocarditis. Because replicate-level Ct values were unavailable, these summaries are descriptive and no significance claims were re-estimated.

## 4 Discussion

The revised analysis changes the central interpretation of the study. Cervical tissues expressed angiogenic, inflammatory, endothelial, exhaustion, and TGF-*β*/fibrotic programmes, and TCGA tumours showed substantial coexpression among several of them. However, with biological specimens as the unit of analysis, no prespecified single-cell programme or ligand–receptor expression product was significantly higher in malignant specimens after multiplicity correction. More importantly, none of the public cohorts contained treatment exposure or CVT outcomes. These data therefore cannot identify a CVT-susceptibility mechanism.

The absence of corrected specimen-level significance is itself informative. Several programme scores were numerically higher in malignant specimens, and the nominal T-cell-exhaustion result was compatible with established cervical-TME biology, but nine specimens provide limited resolution for separating disease-stage effects from interindividual variability. Treating tens of thousands of cells as independent replicates would have produced extremely small P values while overstating the amount of patient-level evidence. By retaining the specimen as the unit of replication, the revised analysis favours valid uncertainty over apparent precision. These results should therefore be viewed as effect estimates for planning larger studies rather than as proof that the programmes are absent or unimportant.

This boundary resolves the compartment problem raised during review. A cervical CAF may produce VEGFA while a cervical endothelial cell expresses KDR, but that observation belongs to the tumour stroma. It does not demonstrate a comparable cardiac sender–receiver pair, and a CAF-to-endothelial relation should not be mapped onto myocardium. Similarly, tumour IL1B–IL1R1 or LGALS9–HAVCR2 expression cannot establish the immune-cell states that distinguish ICI-myocarditis cases. In a directly relevant patient study, Zhu and colleagues compared ICI-treated patients with and without myocarditis and identified expanded, clonally activated cytotoxic CD8+ Temra cells (16). That design illustrates the outcome-linked evidence required for susceptibility inference.

The bevacizumab experiment also requires a different interpretation from the source manuscript. Exogenous VEGFA increased several endothelial transcripts, and bevacizumab reduced them. This is an expected pharmacological neutralisation result, not a paradoxical demonstration of toxicity. Clinical hypertension from VEGF-pathway inhibition is generally linked to systemic loss of endothelial and renal VEGF signalling, including impaired nitric-oxide-mediated vasodilation and microvascular effects (8–11). The present 24-h HUVEC assay began with supraphysiological ligand stimulation and measured mRNA only; it did not model basal VEGF withdrawal, blood pressure, vascular resistance, renal injury, thrombosis, or chronic exposure.

The corrected second cell model is HAEC. IL-1*β* induced inflammatory transcripts and anakinra attenuated several of them, showing that the IL-1 receptor pathway is pharmacologically responsive in aortic endothelium. Because no cardiomyocyte was used, the experiment cannot validate cardiac injury. Nor was ICI exposure included. The result may be useful as endothelial pathway context, but it should not be linked causally to ICI myocarditis.

The TCGA analysis provides an independent view of tumour biology. Strong angiogenesis–TGF-*β*/fibrosis and endothelial-activation–inflammation correlations suggest that these programmes often coexist within cervical tumours. Yet correlation of gene panels in bulk tumour cannot reveal whether the same patients develop treatment toxicity. Labelling TCGA tumours as ‘high CVT risk’ would create an outcome that does not exist in the data. We therefore removed the composite score, ROC curves, odds ratios, incidence estimates, and clinical monitoring recommendations derived from it.

This distinction does not make the cervical TME clinically irrelevant. Tumour vascularity, inflammatory state, and immune composition may influence drug delivery, antitumour response, circulating mediators, and the intensity or duration of treatment exposure. They could therefore be evaluated as candidate modifiers in an outcome-annotated study. The appropriate model would place tumour features alongside established host variables—age, hypertension, diabetes, renal function, prior cardiovascular disease, previous radiotherapy, concomitant chemotherapy, and treatment dose—rather than treat tumour expression as a direct surrogate for myocardial biology. Such a model would also require internal validation, calibration, and an independent cohort before any monitoring strategy could be recommended.

A definitive follow-up study should enrol patients before bevacizumab and/or ICI, prespecify a CVT definition using contemporary cardio-oncology criteria (7), and compare treated CVT-positive with treated CVT-negative patients. Baseline cardiovascular covariates, therapy and dose, blood pressure, troponin, natriuretic peptides, ECG, and imaging should be collected longitudinally. Molecular sampling should prioritise blood and, when clinically available, cardiac-relevant material; matched tumour profiles could then be tested as candidate predictors rather than assumed to be cardiac surrogates. Discovery and validation cohorts are needed, with patient—not cell—as the statistical unit.

This study has substantial limitations. GSE208653 contains only nine specimens, including one ADC, so exact tests have low power and condition is partly confounded with specimen. Broad marker-panel annotation may misclassify fine cell states. The ligand–receptor expression product is descriptive and does not infer signalling. GSE208654 has one specimen per histology and the supplied matrices lack spatial coordinates. TCGA-CESC contains tumour expression but no therapy-linked CVT. No cardiac tissue or CVT-positive/CVT-negative cohort was available. The endothelial assays measure selected mRNAs after short-term stimulation; they include neither cardiomyocytes nor ICI treatment, and the supplied archive lacks replicate-level Ct values, primer sequences, protein measurements, and functional vascular endpoints. These limitations preclude clinical prediction, causal inference, or preventive-treatment recommendations.

## 5 Conclusion

The cervical TME contains angiogenic, endothelial, inflammatory, exhaustion, and TGF-*β*/fibrotic transcriptional programmes that overlap with biological pathways relevant to cardio-oncology. In this small all-specimen reanalysis, none of the prespecified single-cell programmes or ligand–receptor expression products was significantly higher in malignant cervical specimens after multiple-testing correction. TCGA-CESC confirmed intratumoural coexpression but offered no CVT endpoint. HUVEC and HAEC experiments demonstrated pathway responsiveness and receptor-directed blockade, not cardiac toxicity. CVT susceptibility can only be tested in outcome-annotated treated patients, ideally comparing CVT-positive and CVT-negative groups with cardiac-relevant or circulating biospecimens.

## Data Availability

The datasets analysed in this study are publicly available. The single-cell RNA-sequencing and spatial transcriptomic datasets are available in the Gene Expression Omnibus under accession numbers GSE208653 and GSE208654, respectively. TCGA-CESC primary-tumour data are available through the UCSC Xena GDC hub.
